# The feasibility and applications of non-invasive cardiac output monitoring, thromboelastography and transit-time flow measurement in living-related renal transplantation surgery: results of a prospective pilot observational study

**DOI:** 10.1186/2047-1440-3-16

**Published:** 2014-08-29

**Authors:** Stephen J Goodyear, James Barnes, Caitlin E Imray, Robert Higgins, For T Lam, S Habib Kashi, Lam C Tan, Christopher HE Imray

**Affiliations:** 1 University Hospitals Coventry and Warwickshire NHS Trust, Clifford Bridge Road, Coventry CV2 2DX, UK; 2 University of Sheffield Medical School, Beech Hill Rd, Sheffield, South Yorkshire S10 2RX, UK; 3 Warwick Medical School, University of Warwick, Gibbet Hill Rd, Coventry CV4 7AL, UK

**Keywords:** Non-invasive cardiac output monitoring, NICOM, Transit time flow monitoring, TTFM, Thromboelastography, TEG, Living-related, Renal transplant, Delayed graft function, DGF, Thrombosis

## Abstract

**Introduction:**

Delayed graft function (DGF) remains a significant and detrimental postoperative phenomenon following living-related renal allograft transplantation, with a published incidence of up to 15%. Early therapeutic vasodilatory interventions have been shown to improve DGF, and modifications to immunosuppressive regimens may subsequently lessen its impact. This pilot study assesses the potential applicability of perioperative non-invasive cardiac output monitoring (NICOM), transit-time flow monitoring (TTFM) of the transplant renal artery and pre-/perioperative thromboelastography (TEG) in the early prediction of DGF and perioperative complications.

**Methods:**

Ten consecutive living-related renal allograft recipients were studied. Non-invasive cardiac output monitoring commenced immediately following induction of anaesthesia and was maintained throughout the perioperative period. Doppler-based TTFM was performed during natural haemostatic pauses in the transplant surgery: immediately following graft reperfusion and following ureteric implantation. Central venous blood sampling for TEG was performed following induction of anaesthesia and during abdominal closure.

**Results:**

A single incidence of DGF was seen within the studied cohort and one intra-operative (thrombotic) complication noted. NICOM confirmed a predictable trend of increased cardiac index (CI) following allograft reperfusion (mean CI - clamped: 3.17 ± 0.29 L/min/m^2^, post-reperfusion: 3.50 ± 0.35 L/min/m^2^; *P* < 0.05) mediated by a significant reduction in total peripheral resistance. Reduced TTFM at the point of allograft reperfusion (227 ml/min c.f. mean; 411 ml/min (95% CI: 358 to 465)) was identified in a subject who experienced intra-operative transplant renal artery thrombosis. TEG data exhibited significant reductions in clot lysis (LY30 (%): pre-op: 1.0 (0.29 to 1.71), post reperfusion 0.33 (0.15 to 0.80); *P* = 0.02) and a trend towards increased clot initiation following allograft reperfusion.

**Conclusions:**

Reduced renal arterial blood flow (falling without the 95% CI of the mean), was able to accurately predict anastomotic complications within this pilot study. TEG data suggest the emergence of a prothrombotic state, of uncertain clinical significance, following allograft reperfusion. Abrogation of characteristic haemodynamic trends, as determined by NICOM, following allograft reperfusion may permit prediction of individuals at risk of DGF. The findings of this pilot study mandate a larger definitive trial to determine the clinical applications and predictive value of these technologies.

## Introduction

Renal transplantation is the gold standard form of renal replacement therapy [1], with highly significant survival and economic benefits over haemodialysis and peritoneal dialysis.

Aside from acute rejection, the most important post-operative sequela following transplantation is delayed graft function (DGF). There is no unified definition for DGF; indeed, a recent systematic review demonstrated over 18 published definitions [2]. However, DGF is generally defined in the context of the need for dialysis post-transplant, and/or a proportional or absolute inadequate fall in serum creatinine level at a set time point after transplantation. Although rates of DGF in live-donor transplantation are lower than in deceased-donor recipients, the published incidence can be as high as 15%, with an associated doubling in the risk of acute rejection and potential reduction in overall graft survival duration [3,4].

The underlying causes of DGF are complex and multifactorial, but are essentially due to warm ischaemic injury, hypothermic injury and reperfusion injury. In the context of live donation, where warm ischaemic time and cold storage duration are short, minimisation of reperfusion injury is the primary target for amelioration of DGF. The importance of systolic and diastolic blood pressure at the time of reperfusion of renal transplants is well established [5,6]. Furthermore, several vasodilatory therapeutic interventions have been shown to improve DGF both clinically and experimentally [7].

Identification of recipients at risk of developing DGF allows modification of immunosuppressive regimens by sparing or minimisation of calcineurin inhibitors, and use of thymoglobulin and mycophenolate. There is evidence that these measures can significantly reduce the impact of DGF [7-9].

Cardiac output (CO) monitoring during surgical procedures offers a means of ensuring adequate systemic perfusion and is superior to the observation of basic cardiovascular parameters, pulse rate and blood pressure, alone [10]. Real-time non-invasive assessment of CO allows the anaesthetist to utilise a range of inotropic and vasoactive medications intra-operatively to optimise the cardiovascular status of the transplant recipient, thereby maintaining adequate renal arterial blood flow to the allograft following reperfusion and avoiding the risks of Swann-Ganz catheterization [11].

### Cardiac output

Non-invasive cardiac output monitoring (NICOM) based on the principles of bioimpedance plethysmography and more recently bioreactance plethysmography, has been used to provide risk-free real-time data (avoiding the potential morbidities conferred by invasive techniques like Swann-Ganz catheters and oesophageal Doppler monitoring) [12]. For bioreactance based NICOM™ (Cheetah Medical Inc, Portland, Oregon 97201, USA), sensors are placed on the patient’s chest to deliver a low voltage alternating electrical current of known amplitude and frequency across the thorax.

Bioreactance NICOM™, has been validated against invasive Swann-Ganz CO monitoring without the significant associated risks [13].

### Renal blood flow

Renal blood flow (perfusion) may be measured directly by Doppler-based transit-time flow monitoring (TTFM) intra-operatively. A similar implantable technology has been shown to be a feasible technique for identifying at-risk organs in the post-operative period, potentially saving precious kidneys and reducing the frequency of unnecessary explorative procedures [14].

### Thromboelastography

Thromboelastography (TEG) has been proven to be of significant value in the prediction of hyper- and hypo-coagulable states for patients undergoing hepatic transplantation and coronary artery bypass graft surgery, in addition to having an established role in the monitoring of patients receiving intravenous heparin [15]. However, its role is as-yet undefined in the prediction of clotting-related complications of living-related renal transplantation, which may range from severe haemorrhage to thrombosis and failure of the renal allograft. TEG assessment of living related transplant recipients on induction may thus confer significant advantages in the context of diagnosis and management of occult clotting defects. A growing body of evidence suggests that TEG is superior to routine laboratory tests in guiding intra-operative coagulation management [16].

## Aim

This pilot study aims to assess the potential applicability of perioperative non-invasive cardiac output monitoring, transit-time flow monitoring (TTFM) of the transplant renal artery and pre-/perioperative thromboelastograph (TEG) in the early prediction of DGF and perioperative complications for living-related renal transplantation. Positive results arising from this study will form the basis or justification for future prospective research in this important area.

## Methods

Ethical approval for this pilot study was granted by the Black Country Research Ethics Committee and NHS/HSC Research and Development offices. Informed consent for participation in the study was granted by all subjects, there were no refusals of consent.

All living-related transplant recipients were considered for the study. HLA-incompatible allograft recipients were not excluded from the study due to proven excellent outcomes achieved using a previously described immunosuppressive regimen [17]. University Hospitals Coventry and Warwickshire (UHCW) NHS Trust practices parallel laparoscopic donor nephrectomy and retroperitoneal exposure (of the recipient’s external iliac artery and vein), in adjacent theatres, for all living-related renal transplantation. Each surgical team (donor and recipient) comprised a combination of two consultant surgeons, or a single consultant and an experienced registrar/associate specialist. As a consequence, warm ischaemic time was negligible for the donated organs, limited to the time required for extraction of the kidney immediately following stapling of the vascular pedicle. The harvested organ was then immediately received by a member of the transplant team, packed in ice and perfused with cold histidine-tryptophan-ketoglutarate (HTK) solution prior to workbench preparation and transfer into the adjoining theatre for transplantation. Cold ischaemic time was <2 hours for all studied cases.

Pre-determined exclusion criteria for this study included individuals receiving allografts with multiple renal arteries and cases where laparoscopic donor nephrectomy was converted to open nephrectomy (in lieu of prolonged warm ischaemic time). Only one individual was prospectively excluded from the study due to known duplex transplant renal arteries.

Ten consecutive living-related renal transplantation recipients were prospectively studied at UHCW NHS Trust. All patients received immunosuppression with tacrolimus 0.15 mg/kg/day (2 days before transplant), azathioprine 1.5 mg/kg, or mycophenolate 1000 mg bd started on the day of transplant or at the start of pre-transplant plasmapheresis. Methylprednisolone 500 mg and basiliximab 20 mg were administered to all subjects intra-operatively. At surgery, allograft vascular anastomoses were to the external iliac vein and artery in all cases. No modifications to the anaesthetic or surgical technique were made as a consequence of participation in this study.

The results of all pre-operative haematology, biochemistry and radiological investigations (for 24 h prior to surgery) were collected from the Trust’s Clinical Results Reporting System (CRRS) software. Similarly, postoperative data were recorded for a period of 7 days.

Non-invasive cardiac output monitoring (NICOM™; Cheetah Medical Incorporated, Vancouver, WA, USA) was applied following induction of anaesthesia and left *in situ*, actively recording, until the subject was discharged from theatre recovery to the transplant surgical ward.

Transit-time flow measurement of the transplant renal artery was performed using a Doppler flow probe (Veri-Q; Medi Stim ASA, Oslo, Norway). Measurements were performed by the principal surgeon, during two natural intra-operative haemostatic pauses; immediately following allograft reperfusion and after cystoureteric anastomosis.

For the purposes of this pilot study, the development of DGF was defined as the requirement for dialysis within seven days of allograft transplantation.

### NICOM™

Bioreactance is often compared with bioimpedance, an older technology used to determine cardiac output. Bioreactance and bioimpedance are conceptually similar to the principles of frequency modulation (FM) and amplitude modulation (AM) radio. With FM radio, signal detection is based on changes in signal frequency rather than changes in signal amplitude, allowing for greater fidelity in the obtained signal, as amplitude decays exponentially with distance from source, whereas frequency remains constant within a given medium. FM enables significant advantages in filtering noise, for example noise coming from other electronic or physiologic emitters. This is why FM-based systems offer superior performance compared to AM [18].

Prior to the induction of anaesthesia, the NICOM™ device was programmed with the patient’s unique identifying number, age (yrs), sex, height (m) and weight (kg), facilitating calculation of body surface area (BSA; m^2^). Measured haemodynamic data (pulse rate, cardiac output/index) were constantly monitored throughout surgery and recovery (data points automatically recorded at 1-min intervals). Non-invasive blood pressure (NIBP) monitoring was set to measure every 5 minutes, allowing calculation and collection of total peripheral resistance (TPR) and TPR index (TPRI) at equivalent intervals.

Non-invasive cardiac output monitoring was applied by a trained investigator immediately upon entering the operating theatre, prior to the commencement of surgery. The adhesive pre-wired sensors were placed as per device instructions-for-use and in addition to routine anaesthetic monitoring equipment.

### Transit-time flow measurement

Renal arterial blood flow (RBF) may now be directly measured intra-operatively using a validated, Doppler-based transit time flow measurement (TTFM) device (VeriQ, Medi-Stim, Oslo, Norway). Transit time is measured as the time spent by an ultrasound signal that travels between two synchronously placed piezoelectric crystals via a metallic reflector bracket situated on the opposite side of the arterial wall. Blood volume flow (ml/min) calculation is derived from the time difference between ultrasound signals travelling with and against the direction of blood flow within the vessel. This method has been validated against direct measurements of blood flow [19].

The appropriate size of the probe was determined by measurement of the transplant arterial diameter. The probe was placed proximally on the transplant renal artery by the operating surgeon. Flow velocity readings (ml/min) and pulsatility index (PI) data were subsequently recorded, by a named investigator, at 15-sec intervals for a period of 3 minutes. Two datasets were collected for each patient: immediately following graft reperfusion and following cystoureteric anastomosis.

### Thromboelastography

A pre-operative central venous 5-ml blood sample was drawn prior to the commencement of surgery for the purposes of thromboelastographic analysis and immediately processed by a trained senior perfusionist. A post-reperfusion central venous 5-ml blood sample was similarly drawn and processed (at the point of abdominal closure, in each case) for comparison.

### Follow-up

One-year follow-up data including graft failure (a return to dialysis dependence), serum creatinine levels, haemorrhagic and thrombotic graft complications, incidences of rejection and venous thrombo-embolism were recorded and analysed in the context of NICOM™, TTFM, TEG and phlebotomy data findings. Data were collected from CRRS and the patient case notes (including clinic and discharge letters over the 1-year period).

### Statistical analysis

Data were tabulated in Microsoft Excel (Microsoft, CA, USA) spreadsheets and data analysis performed using GraphPad 4 (GraphPad, La Jolla, CA, USA) Statistical Software. Numerical data were assessed using a D’Agostino and Pearson omnibus normality test and the appropriate parametric/non-parametric comparative tests selected (indicated within the text). Categorical data were assessed by Fisher’s exact test. Specific data describing cardiac index (CI) modulation following reperfusion utilized a predetermined *post-hoc* analysis of the NICOM™ data, including CI data-points for 10 minutes pre- and 10 minutes post-allograft reperfusion. Data describing SBP, TPR and TPRI (collected automatically at 5-minute intervals) modulation at reperfusion similarly utilized a predetermined post-hoc analysis of the NICOM™ data, permitting use of the immediately pre-reperfusion and post-reperfusion NIBP values. Significance was attributed at *P* <0.05.

Power calculations for prospective definitive studies were performed by a Z-test, utilising the statistical data from this pilot study.

## Results

Ten appropriate consecutive patients (P1 to P10) undergoing living-related renal transplantation were studied. Demographic data are shown in Table 1.

**Table 1:** Demographic data for ten consecutive living-related donor renal transplant patients (P1 to P10) studied

**Patient**	**Age (yrs)**	**Height (cm)**	**Weight (kg)**	**Body surface area (m**^**2**^**)**	**Body mass index (kg/m**^**2**^**)**	**Ethnicity**	**Dialysis status**	**Previous transplant**	**CMV (donor/recipient)**	**ESRF cause**
P1	30	161	64	1.67	24.7	White European	HD^b^ (fistula)	N	-/+	Henoch-Schonlein Purpura
P2	45	177	101	2.18	32.2	White European	Pre-dialysis	N	-/-	?Hypertensive nephropathy
P3	41	179	77	1.96	24.0	White European	Pre-dialysis	N	-/-	FSGS^c^
P4	24	157	50.4	1.49	20.4	White European	Pre-dialysis	N	+/+	?Hypertensive nephropathy
P5^a^	41	157	66.4	1.67	26.9	South Asian	HD^b^ (vascath)	N	-/+	DM nephropathy
P6	44	185	83	2.07	24.3	White European	HD^b^ (fistula)	Y	-/-	Hypertensive (prev. traumatic nephrectomy)
								Cadaveric ×2 (1993/2010)		
								^d^AIT		
P7	58	168	58.8	1.67	20.8	South Asian	HD^b^ (fistula)	N	+/+	Membranous nephropathy
P8	23	161	49.6	1.5	19.1	White European	CAPD	N	-/-	Chronic pyelonephritis
P9	48	169	77.4	1.88	27.1	White European	HD^b^ (fistula)	N	-/-	IgA nephropathy
P10	37	189	90.6	2.18	25.4	White European	Pre-dialysis	N	-/-	IgA nephropathy

^a^P5 (highlighted) demonstrated DGF at 7-days post-operatively. ^b^HD, haemodialysis; ^c^FSGS, focal segmental glomerulosclerosis; ^d^AIT, antibody incompatible transplantation.

Of the individuals studied, one incidence of DGF was noted (10%; P5). One intra-operative complication (10%; P3; thrombus at the arterial anastomosis, requiring thrombectomy and revision of the anastomosis) identified. One patient was postoperatively commenced on anti-rejection therapy (anti-thymoglobulin antibody) (10%; P7; within the period of follow-up, but did not demonstrate DGF).

### Applicability

NICOM™ monitoring was successfully achieved in 100% of the studied subjects using the agreed-upon protocol. NICOM™ haemodynamic monitoring was subject to sensor-interference only due to prolonged use of coagulation monopolar diathermy during operative (retroperitoneal in all cases) exposure of the iliac vessels. The mean period of data loss during dissection was 5.4 min (95% CI: 2.7 to 8.1) and was consistent with the sensor interference noted by concurrent bioimpedance-based ECG monitoring equipment.

Concomitant use of anaesthetic-monitoring equipment and NICOM™ was best achieved by placing the cranially situated NICOM™ pads laterally to the right upper limb (red) and left upper limb (yellow) adhesive ECG pads. This avoided interference of the bioimpedance-based ECG monitoring. No reciprocal NICOM™ interference was noted in the above orientation.

One episode of NICOM™-monitoring failure (and subsequent data loss; P6) was noted during postoperative patient transfer to the recovery bed. This was due to shearing of a pre-wired NICOM™ sensor lead during transfer, mandating replacement of a pair of adhesive sensor pads and re-calibration of the device (achieved within 12 minutes) to continue monitoring.

TTFM monitoring was achieved in 100% of the studied subjects as per study protocol. The technique was considered technically straightforward, acceptable and repeatable by each of the principle surgeons.

TEG analysis was performed as per protocol in 90% of patients, but was omitted in an individual who did not routinely receive perioperative internal jugular vein (IJV) central venous access.

**Figure 1:**
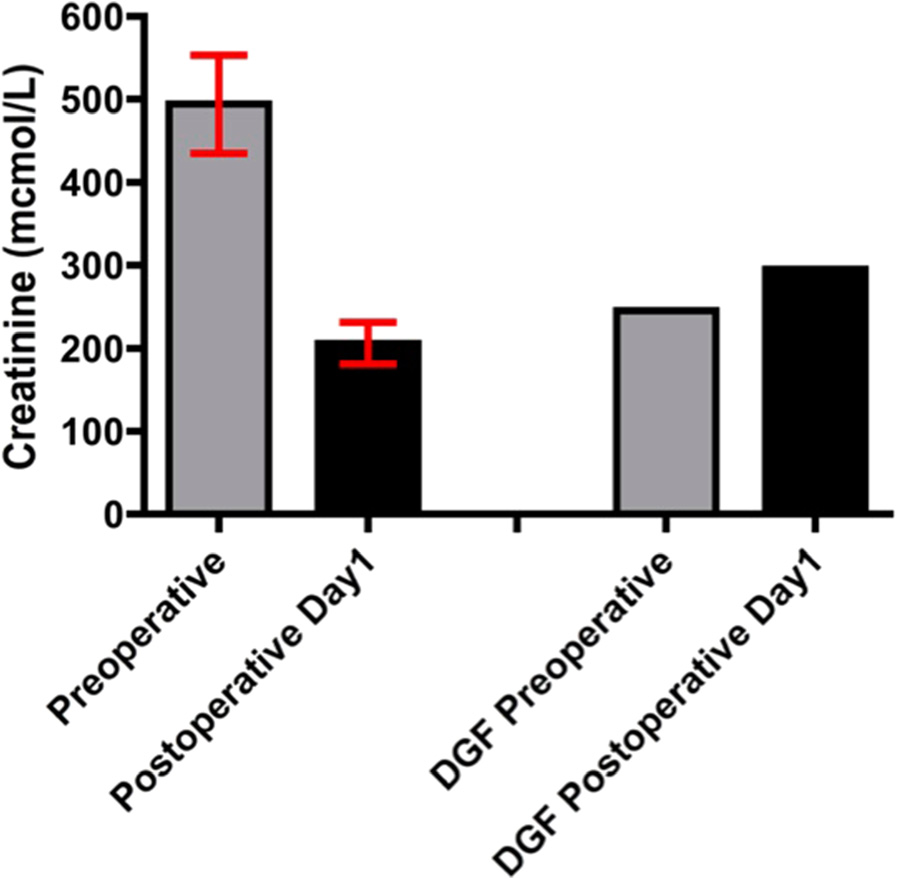
**Pre-operative versus post-operative day 1 serum creatinine.** The left hand pair of bars represent mean (95% CI) creatinine for the ten studied subjects (**P <0.01). The right hand pair of bars represent individual data for patient 5 (P5), who experienced delayed graft function.

**Figure 2:**
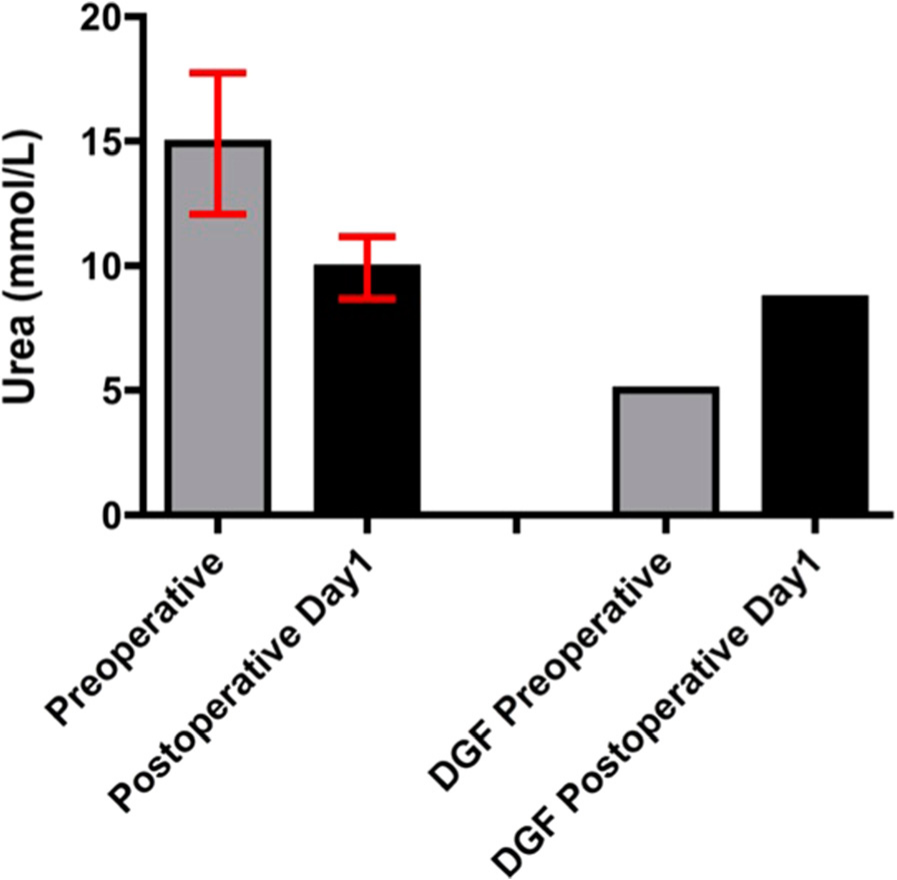
**Pre-operative versus post-operative day 1 serum urea.** The left hand pair of bars represent mean (95% CI) urea for the ten studied subjects (*P* = 0.055). The right hand pair of bars represent individual data for patient 5 (P5), who experienced delayed graft function.

Pre-operative and 24-h postoperative mean blood results are tabulated (Table 2). Patient five (P5) was confirmed to exhibit delayed graft function (DGF), requiring haemodialysis within one-week of transplantation; this subject’s serum creatinine concentration was 243 mcmol/L 7 days P5 exhibited postoperative increases in urea (5 mmol/L to 8.7 mmol/L) and creatinine (245 μmol/L to 295 μmol/L), within 24 h of surgery (Figures 1 and 2), opposing significant biochemical trends shown by non-DGF transplants (Table 2).

**Table 2:** Pre-operative and Postoperative (Day 1) mean values for routine haematological and biochemical investigations of all studied living-related transplant recipients

	**Pre-op**		**Day 1**		**Paired t-test *P *** **value**
	**Mean**	**95% CI**	**Mean**	**95% CI**	
**Na (mmol/L)**	142	140-144	140	137-144	0.315
**K (mmol/L)**	4.9	4.4-5.4	5.1	4.7-5.6	0.190
**Ur (mmol/L)**	14.9	8.5-21.3	9.9	7.1-12.8	*0.055*
**Cr (μmol/L)**	494	361-628	207	150-263	**0.001**
**WCC (10**^**9**^**/L)**	6.3	5.1-7.5	10.4	8.4-12.4	**0.001**
**Hb (g/dL)**	12.1	10.7-13.4	10.12	8.8-11.4	**<0.001**
**Plt (10**^**9**^**/L)**	204	158-249	186	128-243	0.137
**Alb (g/L)**	42	39-46	33	30-37	**<0.001**
**Bili (μmol/L)**	8	6.5-9.8	6.7	4.9-8.5	0.214
**ALT (IU/L)**	120	14-25	17	12-22	0.483
**Ca (adj) (mmol/L)**	2.24	2.08-2.40	2.15	2.01-2.30	**0.013**
**Al Phos (IU/L)**	95	22-168	88	16-160	0.127
**PT (sec)**	11.7	10.9-12.6	12.0	11.3-12.6	1.0
**INR**	1.1	1.0-1.2	1.1	1.1-1.2	1.0
**APTTr**	1.08	1.03-1.12	1.01	0.96-1.06	0.104
**Mg (mmol/L)**	0.87	0.77-0.97	0.74	0.65-0.83	**0.003**
**Phos (mmol/L)**	1.6	1.49-1.76	1.62	1.35-1.89	0.963
**CRP (mg/L)**	8	1-14	19	5-32	**0.032**

Na, sodium; K, potassium; Ur, urea; Cr, creatinine; WCC, white cell count; Hb, haemoglobin; Plt, platelet count; Alb, albumin; Bili, bilirubin; ALT, alanine transaminase; Ca (adj), adjusted calcium; Al Phos, alkaline phosphatase; PT, prothrombin time; INR, international normalized ratio; APTTr, activated partial thromboplastin time ratio; Mg, magnesium; Phos, phosphate; CRP, C-reactive protein.

### NICOM™

Predictable haemodynamic trends were noted during key surgical steps.

**Figure 3:**
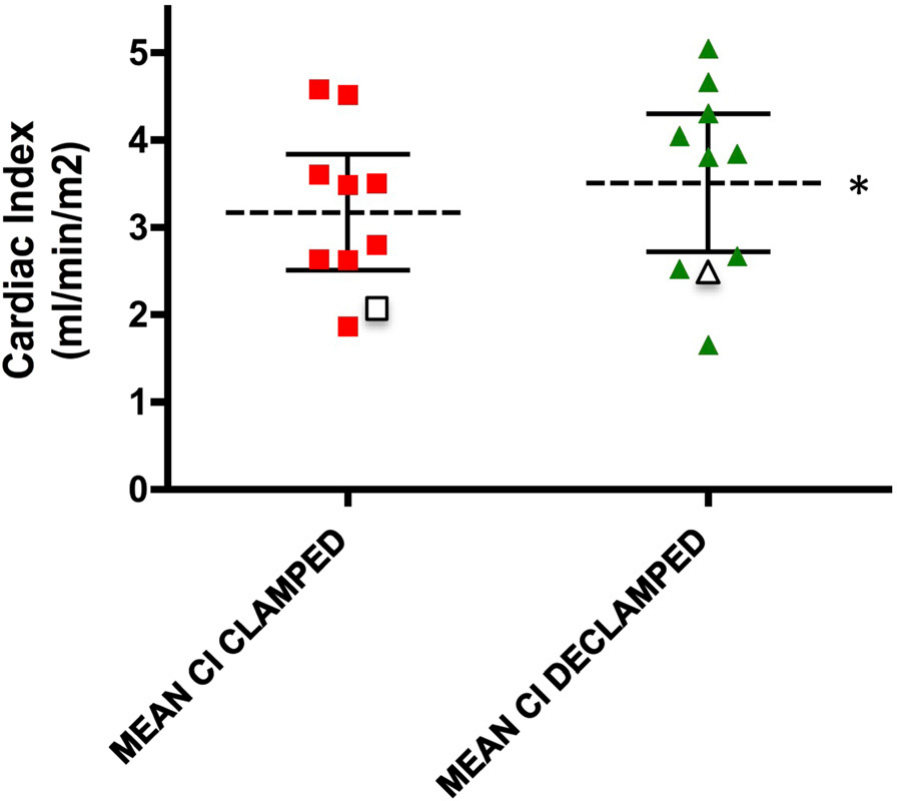
**Cardiac index (CI) during (external iliac artery and vein) clamping and following reperfusion of the allograft (declamping).** The left hand scatter diagram displays the mean (95% CI) cardiac index during vessel clamping for the ten studied subjects. The right hand scatter diagram shows mean (95% CI) cardiac index at reperfusion. (*P <0.05 for mean values). Data for P5, who experienced DGF, are represented by the outlined data points in each diagram.

**Figure 4:**
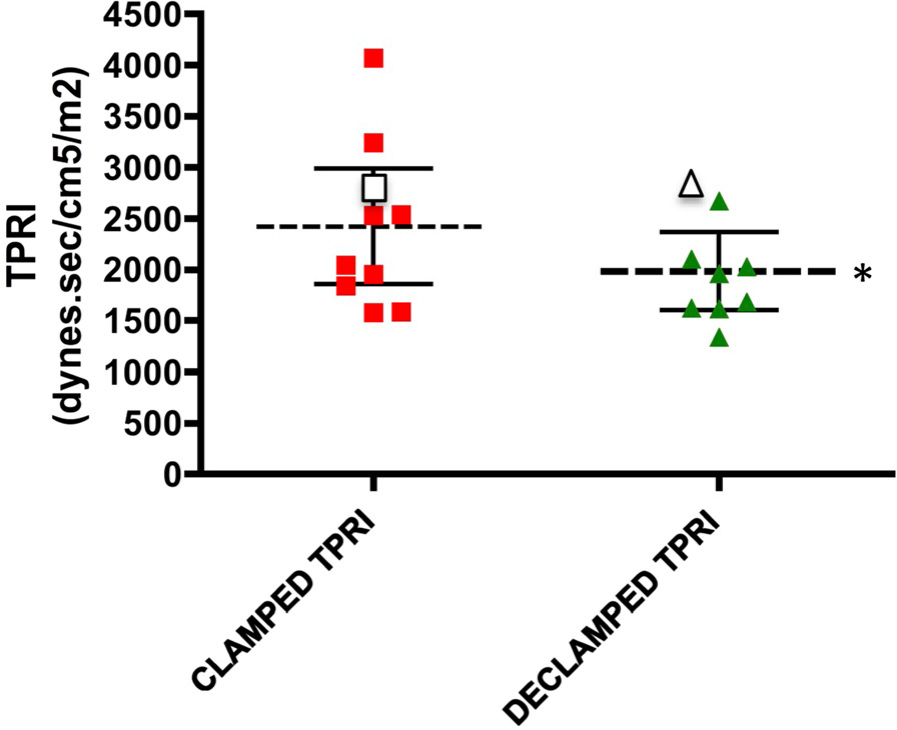
**Total peripheral resistance index (TPRI) during (external iliac artery and vein) clamping and following reperfusion of the allograft (declamping).** The left hand scatter diagram displays the mean (95% CI) total peripheral resistance (TPRI) during vessel clamping for the ten studied subjects. The right hand scatter diagram shows mean (95% CI) TPRI at reperfusion. (*P <0.05 for mean values). Data for P5 who experienced DGF are represented by the outlined data-points in each diagram.

**Figure 5:**
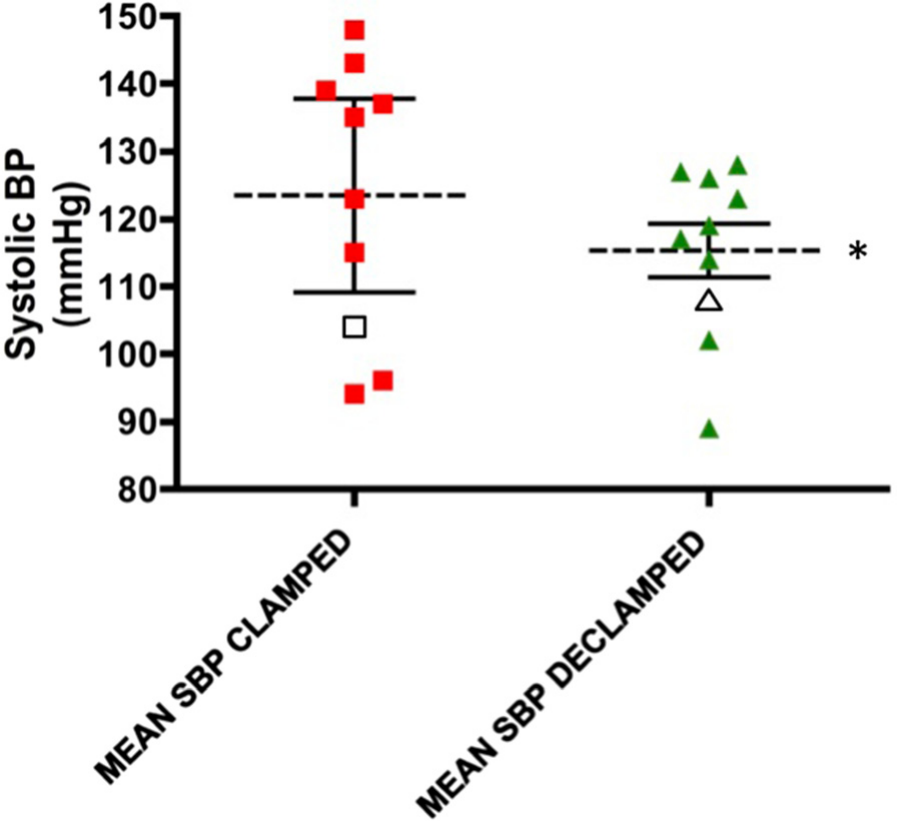
**Mean systolic blood pressure (SBP) during (external iliac artery and vein) clamping and following reperfusion of the allograft (declamping).** The left hand scatter diagram displays the mean (95% CI) SBP during vessel clamping for the ten studied subjects. The right hand scatter diagram shows mean (95% CI) SBP at reperfusion. (*P <0.05 for mean values). Data for P5, who experienced DGF, are represented by the outlined data points in each diagram.

Cardiac index increased following transplant-reperfusion (mean CI - clamped: 3.17 ± 0.29 L/min/m^2^, post-reperfusion: 3.50 ± 0.35 L/min/m^2^; *P* <0.05 - paired t-test; Figure 3) mediated by a reduction in total peripheral resistance index (mean TPRI-clamped: 2240 ± 251dynes.sec/cm^5^/m^2^, post-reperfusion: 1985 ± 166 dynes.sec/cm^5^/m^2^; *P* <0.05 - paired t-test; Figure 4) and associated with a reduction of systolic blood pressure (SBP) (mean -clamped: 126 ± 6.7 mmHg, post-reperfusion: 116 ± 4.3 mmHg; *P* <0.05 - paired t-test; Figure 5).

These characteristic haemodynamic trends (TPRI and SBP) were abrogated in the individual who later experienced delayed graft-function (P5) (Figures 4,5).

### TTFM

**Figure 6:**
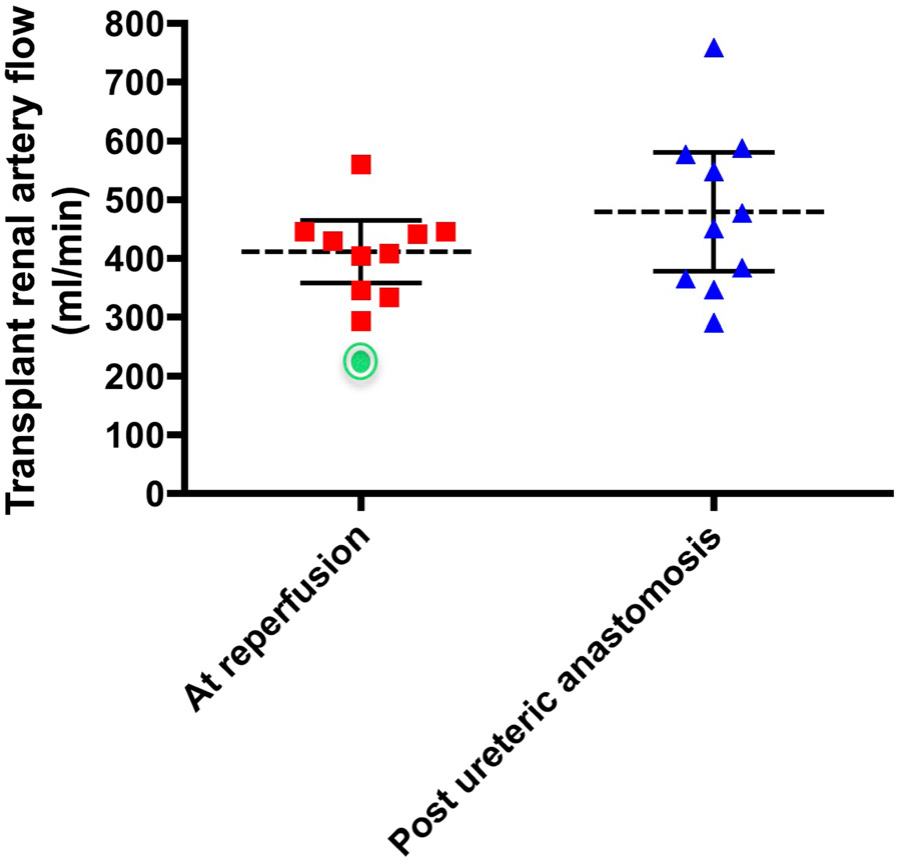
**Mean transplant renal artery flow and 95% CI (left) following allograft reperfusion and (right) following ureteric anastomosis.** The initial data point for P3, who demonstrated anastomotic thrombosis at the point of initial reperfusion, is shown as an encircled point on the left-hand scatter diagram.

Mean transplant arterial blood flow at reperfusion: 411 ml/min (95% CI: 358 to 465) and following cysto-ureteric anastomosis: 480 ml/min (95% CI: 378 to 581; *P* = 0.11 - paired t-test). By comparison, a flow rate of only 227 ml/min was noted in P3; a case of partial transplant arterial thrombosis (Figure 6).

### Thromboelastography

TEG data are displayed in Table 3. All pre-operative TEG values fell within the manufacturer’s stated normal range for clot initiation, propagation and lysis. Comparative paired analysis between pre-operative and post-reperfusion samples for the studied group, however, demonstrated a significant reduction on proportionate clot lysis in the first 30 and 60 minutes (after clot formation: LY30 and LY60 values), suggesting a reduction in fibrinolytic activity toward the end of the surgery, following allograft implantation. There was also a trend (nonsignificant) toward reduced reaction-time (R-value; representative of increased activation of the coagulation cascade, *P* = 0.07) for the post-reperfusion samples.

**Table 3:** **Paired t-test analysis of thromboelastography** (**TEG) values derived from pre-operative and post-reperfusion (paired) samples in renal transplant patients (n = 8)**

	**Pre-operative samples (value, 95% CI)**	**Post-reperfusion samples (value, 95% CI)**	**Paired t-test P value**
**Reaction time (R; min)**	7.0 (6.3-7.8)	6.4 (5.9-6.9)	*0.07*
**Kinetic time (K; min)**	2.10 (1.5-2.7)	2.16 (1.8-2.6)	0.84
**Alpha angle (degrees)**	62.4 (56.6-68.2)	59.5 (52.2-66.8)	0.71
**Maximum amplitude (MA; mm)**	67.4 (61.3-73.6)	65.3 (57.9-72.7)	0.98
**G-value**	11.3 (8.3-14.3)	10.4 (6.6-14.2)	0.84
**LY30 (%)**	1.0 (0.29-1.71)	0.33 (0.15-0.80)	**0.02**
**LY60 (%)**	3.6 (1.7-5.5)	2.0 (0.5-3.5)	**0.02**
**Amplitude (A; mm)**	61.8 (54.4-69.3)	61.6 (53.3-69.8)	0.45
**Coagulation index (CI)**	-0.16 (-1.9-1.59)	-0.25 (-1.7-1.1)	0.52

### Follow-up at 1 year

Graft failure at one year follow-up was limited to one individual (P1: 10% of the studied cohort), a consequence of chronic antibody-mediated rejection onset at 9-months, requiring a return to haemodialysis.

Median creatinine at six months follow-up was 115 μmol/L (interquartile range 93.5 to 131.5) and at one-year follow-up was 118.5 μmol/L (interquartile range 94.0 to 131.0).

Early acute rejection was seen in five subjects (P1, P5, P6, P7 and P9; 50% of studied subjects) and successfully treated in each case with variable immunological regimens (Table 4). There were no significant differences in CI, TPRI or SBP during the clamped and reperfusion (de-clamped) phases of surgery among individuals experiencing acute early rejection and those who did not upon subgroup analysis. Similarly, no detectable differences in renal artery blood flow at reperfusion or following ureteric implantation were evident between these subgroups. Pre-operative thromboelastography demonstrated a trend (nonsignificant) towards increased reaction time (R_(rejection)_ = 5.06 min (95% CI: 4.08 to 6.04) versus R_(no rejection)_ = 2.06 min (95% CI, 1.78 to 5.89); *P* = 0.069, unpaired t-test) and increased clot lysis at 60 minutes (LY60_(rejection)_ = 7.6% (95% CI: 6.14 to 9.06) versus LY60_(no rejection)_ = 6.4% (95% CI: 5.77 to 7.07); P = 0.074, unpaired t-test) for individuals who suffered early acute rejection compared to those who did not. A one-sample Z-test determined that the required study sample size to confirm or refute this observed trend would be nine patients in each arm, a minimum of n = 18 subjects.

**Table 4:** One year follow-up and outcome data for the studied group of ten living-related renal transplant recipients

**Patient**	**DGF**	**Thromboembolic graft complication**		**VTE**	**Haemorrhagic graft complication**		**Rejection**		**Treatment for rejection**	**Creatinine 6 months**	**Creatinine 1 year**	**Dialysis (at 1 yr)**
		**Yes/No**	**Type**		**Yes/No**	**Type**	**Yes/No**	**Type**				
**P1**^**a**^	✗	No		✗	Yes	Large perigraft haematoma	Yes	Early acute rejection.	^b^ATG for early rejection; successful	115	286	Yes
								Chronic antibody mediated rejection from 9 months	^e^Campath, no benefit			
**P2**	✗	No		✗	No		No			120	111	No
**P3**	✗	Yes	Intra-operative transplant artery thrombosis - surgically corrected	✗	No		No			84	87	No
**P4**	✗	No		✗	No		No			67	79	No
**P5**	✓			✗			Yes	Antibody mediated acute rejection cause of ^c^DGF	Early short term dialysis. ATG, Methylprednisolone, ^d^MMF; successful	104	130	No
**P6**	✗	No		✗	No		Yes	Acute early rejection	ATG; successful	134	101	No
**P7**	✗	No		✗	No		Yes	Early antibody mediated rejection	Pulsed oral prednisolone regimen; successful	144	123	No
**P8**	✗	No		✗	No		No			129	129	No
**P9**	✗	No		✗	No		Yes	Early antibody mediated rejection	Pulsed oral prednisolone regimen; successful	103	114	No
**P10**	✗			✗	No		No				132	No

^a^Patient (P) 1 demonstrated graft failure within 1-year as defined by a return to dialysis-dependent status. ^b^ATG, anti-thymocyte globulin; ^c^DGF, delayed graft function; ^d^MMF, mycophenolate mofetil; ^e^Campath, Alemtuzumab.

There were no further incidences of thromboembolic graft complications at one year follow-up and no incidences of venous thromboembolism (VTE) reported.

One patient (P1) experienced a post-operative haemorrhagic complication, requiring the percutaneous drainage of a symptomatic (peri-allograft) retroperitoneal haematoma. The treatment of this was successful, and no further collections developed.

### Sample size calculations

The data contained within this pilot study allows power/sample size calculations to be performed, using the one-sample Z-test, for the purposes of ongoing prospective research.

### NICOM™

A study with 80% power to detect a significant (*P* <0.05) difference in CI between non-DGF and DGF patients would require haemodynamic data for ten patients exhibiting delayed graft function. Based upon the findings of this work (DGF occurred in 10% of patients), and rates of DGF apparent in the current literature (up to 15% of transplants), an n value of 67 to 100 (total) subjects would be required.

However, detection of an equivalent statistical difference in TPRI data would require data for three patients with DGF, equating to a maximum of n = 30 studied subjects.

### TTFM

A study with 80% power to confirm a significant (*P* <0.05) difference in renal artery blood flow between post-anastomotic measurement and post ureteric-implantation measurement was achievable with n = 10 subjects. However, from this study, one of the 10 studied subjects (10%) was found to have anastomotic complications, requiring surgical revision. The markedly reduced flow rate observed in this individual may be confirmed as statistically significant in a study exhibiting two patients with anastomotic complications and would thus require a minimum of n = 20 subjects.

### TEG

A study with 80% power to confirm a significant (*P* <0.05) difference in reaction time (R-value) between pre-operative and post-reperfusion TEG samples would require n = 21 subjects.

## Discussion

Perioperative NICOM™ monitoring, TEG and intra-operative TTFM, in the context of this study, are feasible adjuncts to living-related renal transplantation surgery, with no apparent or potential detrimental effects to the patient.

Characteristic and significant haemodynamic trends were noted by NICOM™ monitoring at graft reperfusion; an increase in CI was noted concurrently with a reduction in TPRI. Systolic blood pressure was also noted to reduce immediately following allograft reperfusion. For the individual who subsequently developed DGF, an increase in SBP and TPRI was noted following reperfusion, abrogating the established trends. However, a modest increase in CI prevailed in this subject. This pilot study was insufficiently powered to demonstrate significant relationship between changes in TPRI and SBP and the development of DGF, however it supports the need for further research to assess a possible predictive role of such cardiac output monitoring in the intra-operative prediction of susceptible individuals. These findings may also be supported by the measurement of biochemical markers on post-operative day 1; a failure to improve creatinine clearance at this juncture was also apparent for P5, who was later determined to exhibit DGF. Further assessment of this relationship may be of value. These clinical findings may facilitate an early decision to adopt calcineurin inhibitor-sparing immunosuppressive regimens, potentially minimizing the impact of DGF [7-9,20].

TTFM data indicated a median transplant-arterial blood flow of 430 ml/min (95% CI: 351 to 472) following allograft reperfusion. P5 data points fell comfortably within this confidence interval, suggesting that the absolute flow-rate values have no obvious application in the prediction of DGF within the limitations of this pilot study. However, one individual (P3) experienced intra-operative thrombosis of the transplant renal artery (at the anastomosis) and upon assessment, demonstrated an average flow rate of only 228 ml/min (lying outside the lower 95% CI for normal subjects). This reading was consistent with the intra-operative appearance of the allograft at reperfusion and facilitated the decision to immediately revise the anastomosis, perform thrombectomy and ultimately salvage the transplanted kidney. Again, significance cannot be attached to the difference in flow rates described above; however, it mandates further investigation. With a sufficiently powered study, a quantifiable flow rate associated with graft anastomotic complications and thus graft failure may be achieved, as is the case in AV-fistula surgery [21] and CABG [22-24]. This may, in turn, form an evidence base for a guideline of minimum acceptable flow rate in living-related transplant surgery, ultimately salvaging precious donor allografts. From the data of this pilot study, the authors suggest a putative minimum acceptable flow rate at reperfusion of approximately 350 ml/min as a cut-off for anastomotic exploration and revision (that is, outside the lower 95% CI of our pilot data).

Within this pilot study, all pre-operative thromboelastography values relating to the activation of the coagulation cascade, thrombus initiation/propagation and lysis fell within the manufacturer’s ‘normal’ range and implies little or no application for this test in the prediction of individuals susceptible to DGF. The authors would, however, recommend the ongoing use of pre-operative TEG and further, larger, studies of its applicability within this context. At present, no clear data exist to describe the outcomes of individuals with abnormal TEG values prior to renal transplantation and a valid, predictive clinical role for this simple test may yet become apparent. Moreover, paired analysis of pre-operative and post-reperfusion TEG data demonstrated a significant reduction in fibrinolysis and a trend towards increased activation of the coagulation cascade toward the end of the renal transplantation surgery. These findings may be considered consistent with the findings of previous work in kidney/pancreas transplantation [25]. By implication, therefore, a potentially hypercoagulable state may thus arise as a consequence of living-related renal transplantation surgery, although our data do not allow speculation as to whether this results from the overall surgical insult or as a consequence of allograft implantation and reperfusion. Our study protocol did not permit further TEG analysis in the post-operative phase, thus potentially missing evidence of perioperative hypercoagulability. The clinical significance of these data are unclear; therefore, larger prospective studies are required to assess these findings in the context of short- and medium-term thromboembolic outcomes and allograft complications. Modification of the study methodology to include post-operative TEG measurements may further define the extent and significance of a hypercoagulable state, and correlation of these data with the occurrence of VTE or thromboembolic graft complications, may provide a clinical context and relevance to future research.

One year follow-up identified a 10% rate of graft failure, a 10% rate of haemorrhagic complications (retroperitoneal haematoma), in addition to the single known thromboembolic perioperative graft complication (10%) within the studied group. No incidences of VTE were reported.

Early acute rejection occurred in half (50%) of the studied cohort and was successfully managed in all cases using variable immunological regimens. Subgroup analysis failed to identify haemodynamic trends measurable by NICOM or TTFM data, suggestive of a clinically applicable role for these technologies in the prediction of this complication. Pre-operative TEG data suggested a trend towards a subclinical hypocoagulable state, manifested by increased reaction time (decreased clot initiation: *P* = 0.074) and increased clot lysis at 60 mins (increased fibrinolysis: *P* = 0.069) for individuals who subsequently developed early acute rejection. Further definitive studies would be required to confirm the significance of these observations and any possible clinical application.

Power calculations based upon the data of this prospective pilot study suggest that definitive studies assessing the role of NICOM in the perioperative prediction of DGF should recruit a minimum of n = 67 patients.

Similarly, the authors recommend that a minimum of n = 20 subjects would be necessary to appraise the role of TTFM as a quality control tool: predictive of anastomotic failure, utilizing a similar study protocol. However, larger studies would clearly be more descriptive if the goal was to ascertain a minimum acceptable transplant-renal artery flow rate at revascularization, as a quality assurance technique.

A study sample size of n = 21 subjects would be sufficient to definitively assess the presence or absence of a hypercoagulable state following allograft reperfusion by thromboelastography. This number would also permit subgroup analysis at one year follow-up, to determine if a role exists for pre-operative TEG values in the prediction of individuals at risk of early acute allograft rejection and should be pursued.

### Key points

1. This pilot study suggests a potential role for NICOM monitoring in the prediction of individuals likely to suffer DGF following living-related renal transplantation, requiring further investigation.

2. There was no apparent correlation between post-reperfusion transplant renal artery blood flow (by TTFM) and the development of DGF within this pilot study.

3. Transplant renal artery TTFM immediately after reperfusion was significantly reduced in a patient found to have anastomotic complications. Further study may allow quantification of a ‘minimum acceptable’ flow rate, serving as a quality indicator for anastomosis exploration and revision.

4. Pre-operative TEG was not able to predict that an individual would experience subsequent thrombotic complications.

5. TEG demonstrated the presence of a potential hypercoagulable state following graft reperfusion, which requires further investigation.

## Abbreviations

AIT: Antibody incompatible transplantation; AM: Amplitude modulation; ATG: Antithymocyte globulin; AV: Arterio-venous; BP: Blood pressure; BSA: Body surface area; CABG: Coronary artery bypass graft; CMV: Cytomegalovirus; CO: Cardiac output; CI: Cardiac index; DBP: Diastolic blood pressure; DGF: Delayed graft function; ECG: Electrocardiogram; ESRF: End-stage renal failure; FM: Frequency modulation; HSC: Health and social care; HTK: Histidine-tryptophan-ketoglutarate; IJV: Internal jugular vein; MMF: Mycophenolate mofetil; NHS: National Health Service; NIBP: Non-invasive blood pressure (measurement); NICOM®: Non-invasive cardiac output monitoring (Cheetah Medical Inc); PI: Pulsatility index; SBP: Systolic blood pressure; TEG: Thromboelastography; TPR: Total peripheral resistance; TPRI: Total peripheral resistance index; TTFM: Transit-time flow monitoring; UHCW: University Hospitals Coventry and Warwickshire; VTE: Venous thromboembolism; 95% CI: 95% confidence interval.

## Competing interests

The authors declare that they have no competing interests.

## Authors’ contributions

SJG conceived and designed the study, performed data collection, interpretation, analysis, drafting & revision of the manuscript and is responsible for the intellectual content of the study. JB perfomed data collection and revision of the manuscript for intellectual content. CEI performed data collection and revision of manuscript for intellectual content. RH conceived and designed the study, performed data interpretation and revision of the manuscript for intellectual content. FTL performed data collection and revision of the manuscript for intellectual content. SHK performed data collection and revision of the manuscript for intellectual content. LCT performed data collection and revision of the manuscript for intellectual content. CHEI conceived and designed the study, performed data interpretation, drafting, revision of manuscript and is responsible for the intellectual content of the study. All authors read and approved the final manuscript.

## Authors’ information

SJG: MD FRCS. SpR in Vascular/General Surgery, West Midlands Deanery.

JB: MBChB MRCS. SpR in General Surgery, West Midlands Deanery.

CEI: MBChB. FY1 in General Surgery, Yorkshire Deanery.

RH: MD FRCP. Consultant Nephrologist, UHCW NHS Trust.

FTL: MS FRCS. Consultant General and Renal Transplant Surgeon, UHCW NHS Trust.

SHK: MB ChM FRCS. Consultant General and Renal Transplant Surgeon, UHCW NHS Trust.

LCT: MD FRCS. Consultant General and Renal Transplant Surgeon, UHCW NHS Trust.

CHEI: (Professor) PhD FRCS FRCP. Director of Research and Development, Associate Medical Director. Consultant Vascular, Endovascular and Renal Transplant Surgeon. Warwick Medical School and UHCW NHS Trust.

## Collaborators

Dr. Nithya Krishnan (Consultant Nephrologist, UHCW NHS Trust).
